# The Dentist’s Ability to Detect Different Restorative Procedures on Periapical Radiographs—Results from a Reliability Study

**DOI:** 10.3390/ijerph20032619

**Published:** 2023-02-01

**Authors:** Annika Wülk, Theresa Meusburger, Helena Dujic, Reinhard Hickel, Andreas Kessler, Katrin Heck, Jan Kühnisch

**Affiliations:** Department of Conservative Dentistry and Periodontology, University Hospital, LMU, 80336 Munich, Germany

**Keywords:** dental diagnostics, reproducibility, reliability, dental restoration, endodontics, dental implants

## Abstract

(1) Background: This in vitro reliability study aimed to determine the inter- and intra-examiner reliability for the detection of direct fillings, indirect crown restorations, root canal fillings and implants on periapical radiographs. (2) Methods: Fourteen dentists (<2 years of clinical experience = 11; >2 years of clinical experience = 3) participated in this diagnostic reliability study in which included a theoretical and practical educational training prior to data collection. The image set of periapical radiographs (N = 150) was examined in two evaluation rounds by all the dentists. Cohen’s Kappa (CK) and a binary logistic regression model were computed. (3) Results: The inter- and intra-examiner reliability were found to be in almost perfect agreement: direct fillings (1st round 0.859/2nd round 0.844/intra 0.910), indirect crown restorations (0.932/0.926/0.955), root canal fillings (0.920/0.886/0.941) and dental implants (0.994/0.988/0.987). The binary logistic regression model revealed that the “evaluation round” and “dentist’s clinical experience” had no significant influence, but for the “diagnostic category”; small, but statistically significant differences were documented. (4) Conclusions: The reliability for detecting direct and indirect restorations, root canal fillings or implants on periapical radiographs was found to be high in the present reliability study on periapical radiographs.

## 1. Introduction

**Figure 1:**
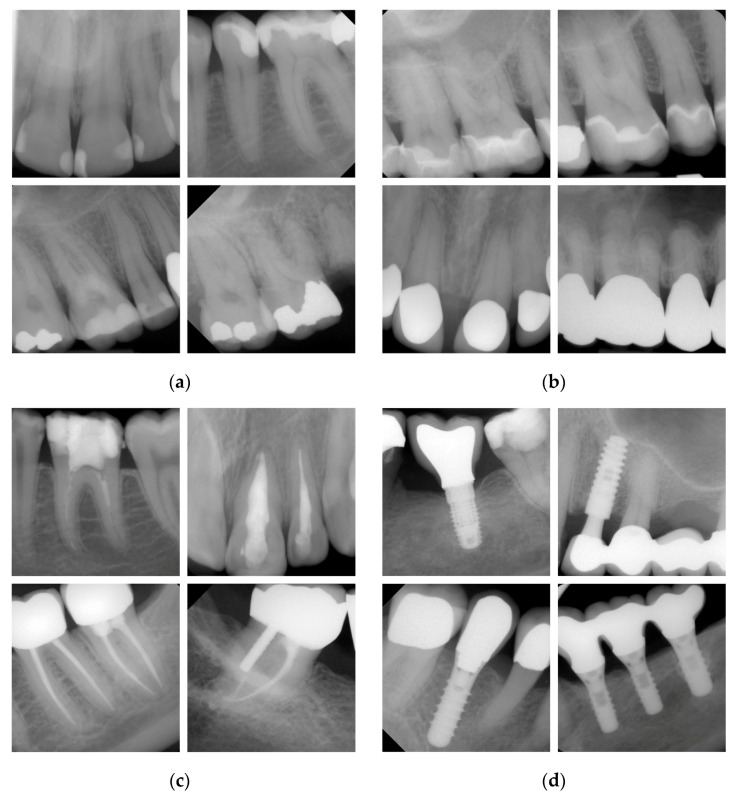
Representative examples of periapical radiographs for the evaluated diagnostic categories: (**a**) directly applied composite or amalgam restorations; (**b**) various types of indirectly manufactured ceramic, porcelain-fused to metal or metal crowns; (**c**) different types of temporary and definitive root canal fillings; (**d**) typical dental implants from divergent manufactures.

Periapical radiographs are frequently used to detect and evaluate different pathologies or restorative procedures in dentistry. In the case of dental restorations, direct fillings (Figure 1a), indirectly manufactured crowns (Figure 1b), root canal fillings (Figure 1c) or dental implants (Figure 1d) must be mainly considered and will appear on X-rays in variant shapes, configurations and different radiopacities according to their clinical specifications and material properties. When conducting diagnostic studies—also under the inclusion of dental restorations on dental radiographs—then, it is important to safeguard an excellent familiarity of all the examiners with the corresponding diagnostic categories. Here, reliability studies are the study type of choice to document the diagnostic outcome between examiners. This approach is well established for the detection of caries, and numerous reliability or reproducibility studies have been conducted in recent decades aimed at assessing mostly new diagnostic criteria and/or detection methods [1,2,3,4,5,6]. In contrast, little information is available about the dentist’s ability to reliably detect different restorative procedures on dental radiographs. Here, only a few reliability studies have been published thus far that have considered the assessment of direct restorations [7], the marginal adaptation of indirect restorations [8] or the presence and quality of endodontic treatments [9,10]. With respect to the limited amount of available information, therefore, this study aimed to determine the inter- and intra-examiner reliability for the detection of direct fillings, indirect crown restorations, root canal fillings and dental implants on periapical radiographs. The hypothesis of this study was that the dentist’s clinical experience and the evaluation round influence the detection of different restorative procedures.

## 2. Materials and Methods

This study on diagnostic reliability was part of the scientific project on „Detection and diagnostics of dental pathologies with artificial intelligence” at the Department of Conservative Dentistry and Periodontology of the Ludwig-Maximilians University (LMU) of Munich. This project was approved by the Ethics Committee of the Medical Faculty of the LMU (project number 20-0798). The reporting of this investigation followed the GRRAS guidelines for reporting reliability and agreement studies [11].

### 2.1. Study Participants

A group of 14 dentists participated in this reliability study. Eleven participants had less than two years of clinical experience; three dentists (all doctors of medicine in dentistry) had worked longer than two years at the university-based dental school. The whole study group took part in theoretical and practical educational training under supervision of the principal investigator (JK) prior to data collection. During the introduction, the study design and diagnostic categories were explained. Furthermore, numerous dental radiographs (N > 500) with frequent and rare pathological findings and restorative procedures were viewed, evaluated and discussed during the training workshop. Subsequently, the same set of periapical radiographs—which was not part of the training course—was analyzed in two evaluation rounds by all the dentists with a minimum interval of 4 weeks to ensure blinding between the diagnostic decisions. All participants were encouraged to perform all the evaluations independently from each other.

### 2.2. Set of Dental Radiographs

For this investigation, a few hundred fully anonymized periapical digital radiographs were primarily selected. All the images were taken at the Department of Conservative Dentistry and Periodontology of the LMU using dental X-ray machines with a 203 mm tube (Heliodent DS, Sirona, Bensheim, Germany), including an X-ray field limitation (30 × 40 mm) and a charge-coupled device (CCD) sensor (Intraoral II, sensor size 30.7 × 40.7 mm, Sirona, Bensheim, Germany). The indication-related exposure time was set to 0.06–0.08 s at a cathode voltage of 60 kV and an amperage of 7 mA. A sensor-holding device (XPP-DS digital sensor holders for Sirona, Dentsply Rinn, Elgin, IL, USA) was available and used if applicable.

The primary selected set of images was screened according to the following inclusion criteria: (1) Each X-ray should present at least one direct restoration, crown, root canal filling or dental implant. Here, no distinction between different types of restoration, root canal filling materials or implant fabricates was made. (2) X-rays from anterior/posterior and upper/lower teeth were selected. (3) The main teeth need to be fully imaged. (4) All the periapical radiographs had to be correctly exposed and should have good contrast and brightness. In the case of distortions, prominent superimposing effects or other qualitative deficiencies, the corresponding X-ray was excluded. Furthermore, any treatment-related radiographs or those to estimate the working length during endodontic therapy were excluded from this reliability study. Finally, 150 periapical radiographs were identified according to the inclusion criteria and a unique identification number was assigned to each image.

### 2.3. Diagnostic Standards

The diagnostic evaluation of all the periapical radiographs used the following standards. Direct restorations, such as composite, cement or amalgam restoration, were diagnosed independently from its location, shape and radiopacity in one category as illustrated in Figure 1a. Indirectly manufactured crowns—detectable as ceramic, porcelain fused to metal or metal crowns—were scored in an additional category (Figure 1b). Further distinctions between restorative materials or insufficiencies [12,13,14] were not made in this study. Root canal fillings were recorded independently from the filling material (Figure 1c). In addition, in this category, no differentiation between a sufficient (full-length, complete and homogenous definitive root canal filling) or an insufficient root canal filling (e.g., over- and underfilling, inconsistencies or voids) was made [15]. The image set further contained images with implants from several manufacturers (Figure 1d). Again, each of the abovementioned categories was chosen when at least one dental finding was detectable. This resulted in a dichotomous decision for each restorative procedure. Again, a distinction between different restorative or root canal filling materials, implant types and qualities was not performed. 

### 2.4. Consensus Decision (Reference Standard)

After completion of the two evaluation rounds, the whole study group re-assessed all the X-rays together during a separate meeting to determine a consensus decision for each periapical radiograph. Here, a dichotomous decision (0 vs. 1) was made again for each of the abovementioned diagnostic categories. If an examiner expressed a divergent finding, the group re-assessed the corresponding X-ray and discussed their points until consensus was reached. This final evaluation served as a reference standard. In total, 101 direct restorations, 63 indirect restorations, 9 root canal fillings and 31 implants were identified.

### 2.5. Data Management and Statistical Analysis

All the data from all the participating dentists (N = 14), two evaluation rounds (N = 2) and the reference standard were entered into an Excel spreadsheet (Excel 2019, Microsoft, Redmond, WA, USA) and checked for plausibility before analysis. Descriptive and explorative data analysis was performed using Excel and SPSS (SPSS Statistics 27, 2020, IBM Corporation, Armonk, NY, USA). The statistical analysis included the calculation of the inter- and intra-examiner reliability among all the participants and the reference standard. For the explorative analysis, Cohen’s Kappa (CK) was computed. To provide an overall value, the arithmetic mean of these estimates was calculated. CK values within the below-mentioned ranges need to be interpreted as follows: 0.00 to 0.20—slight agreement, 0.21 to 0.40—fair agreement, 0.41 to 0.60—moderate agreement, 0.61 to 0.80—substantial agreement and 0.81 to 1.00—(almost) perfect agreement [16,17,18]. In addition, binary logistic regression analysis using a backward elimination model was performed for the outcome (correct/incorrect diagnostic decision in relation to the reference standard). The analysis was adjusted for the diagnostic category, evaluation round and dentist experience (less vs. more than two years of clinical experience).

## 3. Results

The inter- and intra-examiner reliability data in terms of CK can be taken from the corresponding Table for all the participating dentists in relation to the detection of direct fillings (Table 1), indirectly manufactured crowns (Table 2), root canal fillings (Table 3) and implants (Table 4). The mean CK values are summarized in Table 5. In detail, the inter-examiner reliability of the first evaluation round was in an almost perfect agreement range, with the highest CK for dental implants (0.994). The mean CK values for root canal fillings (0.920) and crown restorations (0.932) were nearly the same, followed by direct fillings (0.859). The mean inter-examiner CK values for the first and second evaluation round were found to be of the same order of magnitude (Table 5). The intra-examiner reliability was documented to be excellent throughout all the diagnostic categories (0.910 to 0.987; Table 5).

The level of agreement in comparison to the reference standard indicated only small differences in all four diagnostic categories between the two different evaluation rounds (Table 5). The mean CK values ranged from 0.854/0.837 (direct restorations) to 0.996/0.991 (implants) and were found to be in almost perfect agreement.

The reliability data were further explored by using a binary logistic regression model (Table 6). The results showed that the variables “evaluation round” and “dentist’s clinical experience” had no significant influence on reliability. In contrast, the detection of the included restorative procedure showed statistically significant odds ratios when using implants as a reference. Here, the detection of direct fillings, crown restorations and root canal fillings on periapical radiographs was found to be associated with less reliable diagnostic decisions. 

## 4. Discussion

The aim of this diagnostic study was to determine the inter- and intra-examiner reliability for different restorative procedures—direct and indirect restorations, root canal fillings and implants—on periapical radiographs. In general, the overall documented CK values were in perfect agreement for each diagnostic category (Table 5). In contrast, the binary logistic regression model (Table 6) revealed significant differences. While the evaluation round and the dentist’s clinical experience had no statistical influence on reliability, direct fillings, crown restorations and root canal fillings were detected with a significantly lower reliability in comparison to implants. With respect to this significant difference, the above formulated hypothesis had to be rejected.

When discussing this finding, it should be noted that the documented CK values signal an almost perfect agreement and the recorded deviations between the diagnostic categories were found to be small. However, these differences were tested as significant and should not be overrated. Dental implants are a large-sized and radiopaque structure that can be easily detected on all types of X-rays. Miss-classification might predominantly be caused by documentation errors rather than detection errors. This also applies for metal crowns or amalgam fillings, which show a high radiopacity on X-rays [19]. In contrast, it might be more probable to miss small direct fillings made of composite or gracile temporary endodontic treatments, which are typically less radiopaque than other materials [20].

In daily dental practice, the evaluation of X-rays has to be considered routine; and many different variables have to be screened, detected and, if present, evaluated by the dentist. In contrast, there are surprisingly little scientific data available on how reliable professionals can identify dental restorations, root canal fillings or implants on X-rays. This primarily justified the present study in combination with the aim of determining the diagnostic competence of more or less experienced dentists. With respect to the spectrum of available restoration measures, we prioritized the selection of restoration measures (Figure 1) and further agreed on the dichotomous detection. However, this might be linked to a simpler decision making in comparison to the use of complex classification systems with multiple scores. When considering our results for direct restorations in detail, it must be noted that both the mean inter-examiner CK values amounted to 0.859/0.844 (between all the examiners) and 0.854/0.837 (in relation to the reference standard) and the intra-examiner CK value of 0.910 (Table 5) were found to be high. With respect to the lack of comparable studies, the discussion of the data is subject to restrictions. In a forensic dental study [21], dentists specialized in conservative dentistry reached a high CK value for restoration detection (0.83), followed by dentists specialized in oral surgery (0.64) and dental students in their final semester (0.57). The data support the assumption that there were significant differences between beginners and more experienced practitioners, which were not documented in our study (Table 1 and Table 5). This may show that intensive theoretical and practical educational training prior to data collection had an obviously positive impact on the outcome. This resulted in the fact that the level of knowledge of the young dentists was found to be similar to that of the experienced dentists (Table 6). For indirect restorations, high CK values for inter- and intra-examiner reliability (Table 2 and Table 5) were also observed in the present study. In this context, Mauad et al. [8] investigated the reliability of measuring crown adaptation and found good CK values for inter- (0.633–0.841) and intra-examiner reliability (0.721–0.982), which indicate substantial to perfect agreement between examiners. Unfortunately, we were unable to identify other reliability studies that investigated the dentist’s performance to identify different types of crown restorations.

When viewing, interpreting and discussing the data for the detection of endodontic treatment measures, the previously drawn statements need to be repeated. On the one hand, high CK values for inter- and intra-examiner reliability were registered (Table 3 and Table 5); on the other hand, further work groups documented similar [10,22] or moderate reliability data [9] for the detection of endodontic findings. The latter investigation [9] corroborates the assumption that the interpretation of endodontic information on radiographs may involve errors. The authors highlight the need to train examiners appropriately aiming at improving the data quality. As noted above, the CK values for implant detection (Table 4 and Table 5) achieved the highest agreement compared to all the other categories examined. Again, for this category, no equivalent studies were identified, which hinders comparisons.

Interestingly, no improvement between the first and second evaluation round was observed compared to other studies [22]. This could be addressed by the fact that all the examiners were extensively trained before participating in this study. Furthermore, it must be mentioned that none of the participants were fully inexperienced when agreeing to study participation.

This investigation also had strengths and limitations that need to be discussed. When considering the small number of reliability studies in this field of interest, the relevance of this study project is underlined. The involvement of four diagnostic categories, 14 examiners and 150 periapical radiographs are further strengths of this project. It is noteworthy that the image set represented a broad spectrum of radiological findings that had to be evaluated by participating dentists.

In addition to the mentioned strength, some factors may have had an impact on the examiner’s choice; thus, leading to higher reliability values. It needs to be stated that several inclusion and exclusion criteria were formulated that harmonized the used image set. Therefore, the set of periapical radiographs may not be representative of clinical practice, where often less-than-perfect images need to be assessed. Similarly, a dichotomous decision without performing a thorough classification was made, which may also contribute to the above average reliability. When viewing reliability data from caries categorization studies, it becomes obvious that the CK values are typically found in a range from moderate to excellent reproducibility [3,4]. Likewise, it must be emphasized that only periapical radiographs were used; and therefore, the findings are not fully generalizable for other X-ray projection techniques, e.g., panoramic or bitewing radiographs. Another weakness of the study might be the distribution of diagnostic categories among the set of periapical radiographs, which was not fully balanced over the four selected categories. Variations in the data values may also result from different viewing conditions (lighting, image size and masking of extraneous light). The use of nonstandard monitors for image viewing by the examiners may have affected the radiological diagnosis as well [23,24], but had obviously no influence in the present study, as this variable remained uncontrolled. This matches the typical circumstances in a dentist’s practice where different hardware is omnipresent.

## 5. Conclusions

In result of this diagnostic study, it can be concluded that the reliability for detecting direct and indirect restorations, root canal fillings or implants on periapical radiographs was excellent in terms of Cohen’s Kappa values. Significant differences among the diagnostic categories must be interpreted as small. The dentist’s clinical experience had no influence on the outcome in the present study.

## Figures and Tables

**Table 1:** Inter- and intra-examiner reliability values for the detection of direct restorations between all examiners (N = 14) and in relation to the reference diagnosis (green background color). Gray background colors indicate inter-examiner CK values of the first evaluation round and blue background colors for the second evaluation round. Fields with a white background color represent intra-examiner CK values.

Cohen’s Kappa Direct Restorations			1st Evaluation Round														
		Ref. Stand.	1 *	2	3	4	5	6	7 *	8	9	10	11	12	13	14 *	Mean
	**Ref. Stand.**	1.000	0.698	0.880	0.897	0.850	0.824	0.896	0.910	0.776	0.853	0.880	0.822	0.853	0.897	0.925	0.854
**2nd evaluation round**	1 *	0.723	0.973	0.726	0.768	0.699	0.728	0.740	0.726	0.686	0.755	0.726	0.645	0.755	0.768	0.712	0.726
	**2**	0.852	0.780	0.941	0.956	0.881	0.913	0.926	0.970	0.837	0.913	1.000	0.882	0.942	0.927	0.836	0.901
	**3**	0.866	0.766	0.956	0.971	0.868	0.899	0.912	0.956	0.825	0.899	0.956	0.869	0.928	0.971	0.853	0.897
	**4**	0.767	0.754	0.857	0.842	0.884	0.825	0.867	0.911	0.778	0.825	0.881	0.823	0.854	0.839	0.866	0.840
	**5**	0.810	0.740	0.870	0.884	0.815	0.986	0.869	0.913	0.811	0.943	0.913	0.855	0.971	0.870	0.780	0.868
	**6**	0.879	0.751	0.881	0.896	0.825	0.868	0.925	0.956	0.794	0.898	0.926	0.839	0.898	0.912	0.851	0.876
	7 *	0.940	0.752	0.853	0.837	0.798	0.782	0.850	0.881	0.837	0.913	0.970	0.882	0.942	0.927	0.866	0.905
	**8**	0.852	0.780	0.912	0.927	0.828	0.870	0.911	0.853	0.868	0.840	0.837	0.810	0.840	0.796	0.792	0.806
	**9**	0.838	0.766	0.898	0.912	0.814	0.856	0.897	0.810	0.898	0.928	0.913	0.884	0.943	0.899	0.810	0.880
	**10**	0.796	0.754	0.885	0.899	0.774	0.815	0.855	0.769	0.857	0.843	0.884	0.882	0.942	0.927	0.836	0.901
	**11**	0.820	0.724	0.882	0.897	0.798	0.840	0.880	0.792	0.882	0.839	0.856	0.882	0.855	0.840	0.778	0.834
	**12**	0.896	0.766	0.956	0.971	0.870	0.913	0.925	0.867	0.927	0.912	0.899	0.897	0.956	0.899	0.810	0.891
	**13**	0.767	0.679	0.772	0.786	0.719	0.762	0.798	0.739	0.772	0.789	0.748	0.770	0.817	0.789	0.853	0.879
	14 *	0.910	0.752	0.941	0.956	0.856	0.898	0.940	0.881	0.912	0.897	0.884	0.881	0.985	0.800	0.866	0.819
	**Mean**	0.837	0.751	0.880	0.887	0.812	0.839	0.867	0.814	0.871	0.856	0.834	0.841	0.900	0.765	0.891	0.910

* Dentists with more than 2 years of clinical experience.

**Table 2:** Inter- and intra-examiner reliability values for the detection of crown restorations between all examiners (N = 14) and in relation to the reference diagnosis (green background color). Gray background colors indicate inter-examiner CK values of the first evaluation round and blue background colors for the second evaluation round. Fields with a white background color represent intra-examiner CK values.

Cohen’s Kappa Crown Restorations			1st Evaluation Round														
		Ref. Stand.	1 *	2	3	4	5	6	7 *	8	9	10	11	12	13	14 *	Mean
	**Ref. Stand.**	1.000	0.875	0.973	0.973	0.959	0.946	0.891	0.945	0.917	0.959	0.973	0.986	0.945	0.959	0.986	0.949
**2nd evaluation round**	1 *	0.889	0.957	0.875	0.875	0.834	0.848	0.849	0.847	0.845	0.834	0.875	0.862	0.847	0.861	0.861	0.855
	**2**	0.986	0.902	0.986	1.000	0.959	0.973	0.919	0.973	0.917	0.959	1.000	0.959	0.973	0.986	0.959	0.958
	**3**	0.959	0.874	0.973	0.986	0.959	0.973	0.919	0.973	0.917	0.959	1.000	0.959	0.973	0.986	0.959	0.958
	**4**	0.973	0.861	0.959	0.986	0.986	0.932	0.878	0.959	0.904	0.973	0.959	0.945	0.959	0.945	0.945	0.935
	**5**	0.932	0.875	0.945	0.945	0.932	0.986	0.946	0.973	0.890	0.959	0.973	0.932	0.973	0.959	0.932	0.943
	**6**	0.932	0.848	0.946	0.946	0.932	0.946	0.959	0.919	0.836	0.905	0.919	0.878	0.919	0.905	0.878	0.898
	7 *	0.986	0.874	0.973	0.945	0.959	0.918	0.918	0.931	0.917	0.959	0.973	0.932	1.000	0.959	0.931	0.947
	**8**	0.959	0.845	0.945	0.945	0.959	0.890	0.891	0.945	0.903	0.904	0.917	0.904	0.917	0.903	0.903	0.898
	**9**	0.959	0.847	0.945	0.973	0.986	0.918	0.919	0.945	0.945	0.973	0.959	0.945	0.959	0.945	0.945	0.939
	**10**	0.891	0.805	0.904	0.931	0.918	0.877	0.932	0.877	0.876	0.932	0.918	0.959	0.973	0.986	0.959	0.958
	**11**	0.973	0.889	0.986	0.986	0.973	0.959	0.959	0.959	0.931	0.959	0.918	0.959	0.932	0.945	0.973	0.933
	**12**	0.973	0.889	0.986	0.986	0.973	0.959	0.959	0.959	0.931	0.959	0.918	1.000	0.973	0.959	0.931	0.947
	**13**	0.891	0.779	0.877	0.904	0.918	0.878	0.905	0.904	0.877	0.932	0.918	0.891	0.891	0.887	0.945	0.945
	14 *	0.973	0.861	0.959	0.959	0.973	0.904	0.905	0.959	0.959	0.959	0.891	0.945	0.945	0.891	0.959	0.932
	**Mean**	0.948	0.858	0.946	0.950	0.948	0.919	0.924	0.933	0.918	0.940	0.900	0.950	0.950	0.890	0.932	0.955

* Dentists with more than 2 years of clinical experience.

**Table 3:** Inter- and intra-examiner reliability values for the detection of root canal fillings between all examiners (N = 14) and in relation to the reference diagnosis (green background color). Gray background colors indicate inter-examiner CK values of the first evaluation round and blue background colors for the second evaluation round. Fields with a white background color represent intra-examiner CK values.

Cohen’s Kappa Root Canal Filling			1st Evaluation Round														
		Ref. Stand.	1 *	2	3	4	5	6	7 *	8	9	10	11	12	13	14 *	Mean
	**Ref. Stand.**	1.000	0.938	0.938	0.938	0.882	0.938	0.938	0.938	0.831	0.882	0.938	0.847	0.938	0.938	0.938	0.916
**2nd evaluation round**	1 *	0.938	1.000	1.000	1.000	0.938	1.000	0.868	1.000	0.882	0.938	1.000	0.786	1.000	1.000	1.000	0.955
	**2**	0.938	1.000	1.000	1.000	0.938	1.000	0.868	1.000	0.882	0.938	1.000	0.786	1.000	1.000	1.000	0.955
	**3**	1.000	0.938	0.938	0.938	0.938	1.000	0.868	1.000	0.882	0.938	1.000	0.786	1.000	1.000	1.000	0.955
	**4**	0.790	0.850	0.850	0.790	0.790	0.938	0.813	0.938	0.831	0.882	0.938	0.744	0.938	0.938	0.938	0.901
	**5**	0.938	1.000	1.000	0.938	0.850	1.000	0.868	1.000	0.882	0.938	1.000	0.786	1.000	1.000	1.000	0.955
	**6**	0.944	0.882	0.882	0.944	0.737	0.882	0.882	0.868	0.764	0.813	0.868	0.786	0.868	0.868	0.868	0.845
	7 *	0.938	1.000	1.000	0.938	0.850	1.000	0.882	1.000	0.882	0.938	1.000	0.786	1.000	1.000	1.000	0.955
	**8**	0.831	0.882	0.882	0.831	0.737	0.882	0.786	0.882	0.893	0.944	0.882	0.706	0.882	0.882	0.882	0.860
	**9**	0.938	1.000	1.000	0.938	0.850	1.000	0.882	1.000	0.882	0.938	0.938	0.744	0.938	0.938	0.938	0.910
	**10**	0.938	1.000	1.000	0.938	0.850	1.000	0.882	1.000	0.882	1.000	1.000	0.786	1.000	1.000	1.000	0.955
	**11**	0.730	0.673	0.673	0.730	0.545	0.673	0.696	0.673	0.696	0.673	0.673	0.797	0.786	0.786	0.786	0.773
	**12**	0.938	1.000	1.000	0.938	0.850	1.000	0.882	1.000	0.882	1.000	1.000	0.673	1.000	1.000	1.000	0.955
	**13**	0.868	0.930	0.930	0.868	0.759	0.930	0.813	0.930	0.813	0.930	0.930	0.612	0.930	0.930	1.000	0.955
	14 *	0.938	1.000	1.000	0.938	0.850	1.000	0.882	1.000	0.882	1.000	1.000	0.673	1.000	0.930	1.000	0.955
	**Mean**	0.905	0.935	0.935	0.897	0.798	0.935	0.849	0.935	0.840	0.935	0.935	0.666	0.935	0.870	0.935	0.941

* Dentists with more than 2 years of clinical experience.

**Table 4:** Inter- and intra-examiner reliability values for the detection of dental implants between all examiners (N = 14) and in relation to the reference diagnosis (green background color). Gray background colors indicate inter-examiner CK values of the first evaluation round and blue background colors for the second evaluation round. Fields with a white background color represent intra-examiner CK values.

Cohen’s Kappa Implants			1st Evaluation Round														
		Ref. Stand.	1 *	2	3	4	5	6	7 *	8	9	10	11	12	13	14 *	Mean
	**Ref. Stand.**	1.000	1.000	1.000	1.000	1.000	1.000	1.000	1.000	0.959	1.000	1.000	1.000	1.000	0.980	1.000	0.996
**2nd evaluation round**	1 *	1.000	1.000	1.000	1.000	1.000	1.000	1.000	1.000	0.959	1.000	1.000	1.000	1.000	0.980	1.000	0.995
	**2**	1.000	1.000	1.000	1.000	1.000	1.000	1.000	1.000	0.959	1.000	1.000	1.000	1.000	0.980	1.000	0.995
	**3**	1.000	1.000	1.000	1.000	1.000	1.000	1.000	1.000	0.959	1.000	1.000	1.000	1.000	0.980	1.000	0.995
	**4**	0.979	0.979	0.979	0.979	0.979	1.000	1.000	1.000	0.959	1.000	1.000	1.000	1.000	0.980	1.000	0.995
	**5**	1.000	1.000	1.000	1.000	1.000	1.000	1.000	1.000	0.959	1.000	1.000	1.000	1.000	0.980	1.000	0.995
	**6**	1.000	1.000	1.000	1.000	1.000	1.000	1.000	1.000	0.959	1.000	1.000	1.000	1.000	0.980	1.000	0.995
	7 *	1.000	1.000	1.000	1.000	1.000	1.000	1.000	1.000	0.959	1.000	1.000	1.000	1.000	0.980	1.000	0.995
	**8**	0.958	0.958	0.958	0.958	0.958	0.958	0.958	0.958	0.917	1.000	1.000	1.000	1.000	0.980	1.000	0.976
	**9**	1.000	1.000	1.000	1.000	1.000	1.000	1.000	1.000	1.000	1.000	1.000	1.000	1.000	0.980	1.000	0.998
	**10**	0.979	0.979	0.979	0.979	0.979	0.979	0.979	0.979	0.979	0.979	0.979	1.000	1.000	0.980	1.000	0.998
	**11**	1.000	1.000	1.000	1.000	1.000	1.000	1.000	1.000	1.000	1.000	1.000	1.000	1.000	0.980	1.000	0.998
	**12**	1.000	1.000	1.000	1.000	1.000	1.000	1.000	1.000	1.000	1.000	1.000	1.000	1.000	0.980	1.000	0.998
	**13**	0.958	0.958	0.958	0.958	0.958	0.958	0.958	0.958	0.958	0.958	0.958	0.958	0.958	0.938	1.000	0.982
	14 *	1.000	1.000	1.000	1.000	1.000	1.000	1.000	1.000	1.000	1.000	1.000	1.000	1.000	1.000	1.000	1.000
	**Mean**	0.991	0.990	0.990	0.990	0.987	0.992	0.992	0.992	0.973	0.995	0.982	0.997	0.997	0.961	1.000	0.987

* Dentists with more than 2 years of clinical experience.

**Table 5:** Mean CK values of the inter- and intra-examiner reliability for all evaluated categories.

Variable	Inter- Examiner Reliability	Inter- Examiner Reliability	Intra- Examiner Reliability	Inter- Examiner Reliability	Inter- Examiner Reliability
	1st Round	2nd Round	-	1st Round/Reference Stand.	2nd Round/Reference Stand.
**Direct restorations**	0.859 (0.726–0.905)	0.844 (0.751–0.891)	0.910 (0.789–0.986)	0.854 (0.698–0.925)	0.837 (0.723–0.940)
**Indirect restorations**	0.932 (0.855–0.958)	0.926 (0.858–0.950)	0.955 (0.887–0.986)	0.949 (0.875–0.986)	0.948 (0.891–0.986)
**Root canal filling**	0.920 (0.831–0.938)	0.886 (0.730–1.000)	0.941 (0.790–1.000)	0.916 (0.773–0.955)	0.905 (0.666–0.935)
**Implant**	0.994 (0.959–1.000)	0.988 (0.958–1.000)	0.987 (0.917–1.000)	0.996 (0.976–1.000)	0.991 (0.961–1.000)

**Table 6:** Odds ratios with corresponding 95% confidence intervals (CI) and *p*-values were computed according to the logistic regression model. Bold numbers illustrate a significant influence.

Variables	Groups	Odds Ratio	95% CI	*p*-Value
**Categories**	Implant	1	-	-
	Direct restorations	0.03	0.02–0.06	<0.001
	Crown restorations	0.08	0.04–0.17	<0.001
	Root canal filling	0.21	0.10–0.43	<0.001
**Evaluation round**	1st	1	-	-
	2nd	0.90	0.74–1.09	0.266
**Clinical experience**	≤2 years	1	-	-
	>2 years	1.04	0.82–1.31	0.755

## Data Availability

The datasets generated and analyzed during the current study are available from the corresponding author on reasonable request.
